# Effect of epidural dexmedetomidine in single-dose combined with ropivacaine for cesarean section

**DOI:** 10.1186/s12871-024-02519-4

**Published:** 2024-04-08

**Authors:** Minghao Liu, Xuezi Chen, Dan Guo

**Affiliations:** 1 Department of Anesthesiology, Chengdu Fifth People’s Hospital (The Second Clinical Medical College, Geriatric Diseases Institute of Chengdu/Cancer Prevention and Treatment Institute of Chengdu, Affiliated Fifth People’s Hospital of Chengdu University of Traditional Chinese Medicine), Chengdu, China; 2 Department of Anesthesiology, the First Affiliated Hospital of Chongqing Medical University, Chongqing, China; 3 Department of Ultrasound Imaging, Chengdu Fifth People’s Hospital (The Second Clinical Medical College, Geriatric Diseases Institute of Chengdu/Cancer Prevention and Treatment Institute of Chengdu, Affiliated Fifth People’s Hospital of Chengdu University of Traditional Chinese Medicine), Chengdu, 611137 China

**Keywords:** Cesarean section, Dexmedetomidine, Epidural anesthesia, Pain, Sedation

## Abstract

**Background:**

Dexmedetomidine has arousal sedation and analgesic effects. We hypothesize that epidural dexmedetomidine in single-dose combined with ropivacaine improves the experience of parturient undergoing cesarean section under epidural anesthesia. This study is to investigate the effect of 0.5 µg/kg epidural dexmedetomidine combined with epidural anesthesia (EA) in parturients undergoing cesarean section.

**Methods:**

A total of 92 parturients were randomly divided into Group R (receiveing epidural ropivacaine alone) Group RD (receiveing epidural ropivacaine with 0.5 µg/kg dexmedetomidine). The primary outcome and second outcome will be intraoperative NRS pain scores and Ramsay Sedation Scale.

**Results:**

All 92 parturients were included in the analysis. The NRS were significantly lower in Group RD compared to Group R at all observation timepoint (*P* > 0.05). Higher Ramsay Sedation Scale was found in Group RD compared to Group R (*P* < 0.001). No parturient has experienced sedation score of 4 and above. No significant difference regarding the incidence of hypotension, bradycardia and nausea or vomiting, Apgar scores and the overall satisfaction with anesthesia was found between Group R and Group RD (*P* > 0.05).

**Conclusion:**

Epidural dexmedetomidine of 0.5 µg/kg added slightly extra analgesic effect to ropivacaine in EA for cesarean section. The sedation of 0.5 µg/kg epidural dexmedetomidine did not cause mother-baby bonding deficit. Satisfaction with anesthesia wasn’t significantly improved by epidural dexmedetomidine of 0.5 µg/kg. No additional side effect allows larger dose of epidural dexmedetomidine attempt.

**Trial registration:**

This study was registered at www.chictr.org.cn (ChiCTR2000038853).

**Supplementary Information:**

The online version contains supplementary material available at 10.1186/s12871-024-02519-4.

## Background

Cesarean section is mostly done under general anesthesia (GA) or neuraxial anesthesia. Epidural anesthesia (EA) is one of the preferred neuraxial anesthetic technique for cesarean section, which avoids the risks related to GA (especially failed intubation and aspiration), allows the parturient to maintain favorable early mother-child bonding and has less influence on hemodynamics compared to spinal anesthesia [1–4]. Nevertheless, the main shortcomings of EA resides in inadequate for visceral pain relief during surgery [5] and slow onset of action. The combination of adjuvant (such as sufentanil) and local anesthetics has nowadays became a common method to improve epidural anesthesia [6]. However, there are several side-effects related to neuraxial opioids such as pruritus, nausea and vomiting and utmost vigilance needy respiratory depression which can be life-threatening [7, 8].

Sedation can decrease the need of opioids during regional anesthesia, thus reduce the rate of postoperative nausea and vomiting [9]. Besides, Sedation contributes to patients’ higher satisfaction and increase patients’ acceptance of regional anesthesia [10, 11]. In an obstetrical setting, early skin-to-skin contact between mother and newborn benefits a lot, such as reducing postpartum bleeding rates [12], promoting the release of oxytocin and endorphins [13, 14], thus improve mother’s mood tone [14], while inappropriate sedation might affect skin-to-skin contact [15].

Dexmedetomidine is a highly selective α2 agonist which has arousal sedation effects, analgesic, and anti-sympathetic effects. Compared to intrathecal opioids, the intrathecal use of dexmedetomidine can reduce respiration depression, nausea, vomiting, shivering and other drawbacks associated with opioids [1, 16, 17]. Intrathecally used dexmedetomidine in combination with local anesthetics has been proved to enhance intraoperative anesthetic effects of neuraxial anesthesia and improved maternal satisfaction after cesarean section [18–20]. It’s still not well-understood the effect of combining epidural dexmedetomidine with local anesthetic in cesarean section under epidural anesthesia.

This prospective, randomized, double-blind controlled study was to investigate the effectiveness of epidural dexmedetomidine in combination with ropivacaine on parturients undergoing cesarean section with epidural anesthesia.

## Methods

### Ethics

This study was registered at www.chictr.org.cn (ChiCTR2000038853 **07/10/2020**) and its protocol was accredited by the Ethics Committee of The First Affiliated Hospital of Chongqing Medical University. Participants were well-informed of the study protocol and written informed consent was taken from all the participants present in the study. This study was conducted at The First Affiliated Hospital of Chongqing Medical University, Chongqing, China, between December 2020 and July 2021. The trail was conducted as per the declaration of Helsinki and submitted in the format of CONSORT guidelines.

### Participant recruitment

Parturients who are scheduled for elective cesarean section under EA between December 2020 and July 2021 were eligible for this study. The inclusion criteria for the study were as follows: age between 18 and 39 years old; singleton pregnancy; American Society of Anesthesiologists (ASA) physical status II; ≥ 37 weeks’s gestation. The exclusion criteria of the study were as follows: EA is contraindicated; organ dysfunction such as hypertension, cardiopulmonary disease, placenta previa, fetal distress in utero, and cardiac conduction or rhythm abnormalities; allergy or intolerance to one of the study medications, chronic analgetic use for longer than 3 months; any previous EA or abdominal surgery.

### Randomization and masking

Parturients who were eligible according to criteria were allocated, in a 1:1 ratio, to receive epidural 90 mg ropivacaine (group R) or 90 mg ropivacaine with 0.5 µg/kg dexmedetomidine (group RD) (*n* = 46 parturients per group), via a computer-generated randomization table. The protocol statistician created the randomization schedule. On the day of surgery, a research assistant provided the coinvestigator anesthesiologist with an opaque card containing the randomization details. The unblinded coinvestigator anesthesiologist prepared the solutions but was not involved in data collection. The blinded study anesthesiologist administered the aesthetic procedure and collected the data. Nulliparas remained unaware of their intervention assignment.

### Anesthesia procedure

All parturients were anesthetized with epidural anesthesia and routine epidural puncture was performed at 2 to 3 lumbar interspace with parturients in the right lateral decubitus position. An epidural catheter was inserted 4 cm cephalad into the epidural space. Then parturients were immediately positioned supine with a 15-degree left tilt and received a test dose of 3 mL of 1% lidocaine through the epidural catheter in order to avoid accidental intrathecal or intravascular misplacement. After that, parturients received epidural 90 mg ropivacaine or 90 mg ropivacaine with 0.5 µg/kg dexmedetomidine according to the group allocation via epidural catheter. Effectiveness of EA was defined as bilateral T6-S5 or above sensory block to pinprick within 30 min after injection of study medications. If it was not achieved, additional ropivacaine was administered with the total amount not exceeding 200 mg. Otherwise, EA was turned into GA. An experienced anesthesiologist performed all anesthesia procedures. Surgery started after a T6 level of analgesia was reached and all surgical procedures were performed by the same group of surgeons.

### Monitoring and interventions

After arriving in the operating room, all parturients received standard monitoring including electrocardiography, respiratory rate, pulse oximetry, non-invasive blood pressure monitoring (NIBP), and temperature. A reservoir mask was placed on parturients and oxygen was given at a rate of 4 L/min to all parturients. 5 ml/kg of lactated Ringer’s solution was administrated intravenously to all parturients before anesthesia.

Intraoperative pain severity assessment was done on 0 to 10 numerical rating scale (NRS) with 0 and 10 regarded as “no pain” and “worst imaginable pain” [21]. If parturients suffered from visceral pain of NRS > 4, intravenous 5 µg of sufentanil would be injected after cord clamping and cutting.

Intraoperative sedation was rated by using the Ramsay Sedation Scale [22]: 1, patient anxious and agitated or restless or both; 2, patient co-operative, orientated, and tranquil; 3, drowsiness but can respond to instructions; 4, brisk response; 5, a sluggish response; and 6, no response.

Satisfaction with anesthesia was assessed by using a five-point Likert scale: 1, very dissatisfied; 2, dissatisfied; 3, averagely satisfied; 4, satisfied; and 5, very satisfied. Hypotension, defined as mean arterial pressure (MAP) < 60mmHg or > 20% decline from the baseline BP, was treated with intravenous 50 µg phenylephrine, repeated if needed. Bradycardia was defined as a heart rate (HR) < 60 beats/min and was treated by intravenous atropine 0.25 mg if no hypotension was present, and if hypotension was present with bradycardia, then ephedrine 6 mg was given. Intraoperative respiratory depression was defined as respiratory rate less than or equal to 8 breaths/min or SpO2 < 95% and treated with assisted ventilation.

Timepoint was defined as: T0, before EA; T1, 15 min after EA; T2, fetal delivery; T3, uterus suture; T4, peritoneal closure.

### Data collection

Data collection included parturients demographics (age, weight, height, and gestational age) and intraoperative measures. NRS pain scores was assessed at T2, T3 and T4. Ramsay Sedation Scale, NIBP and HR were collected at T0, T1, T2, T3 and T4. Any episode of side effects such as hypotension, bradycardia, nausea and vomiting were recorded. Neonatal Apgar scores were measured at 1 min and 5 min after fetal delivery. Satisfaction with anesthesia was collected at 3 months after hospital discharge via a telephone follow-up. The primary outcomes were NRS, Ramsay Sedation Scale and satisfaction with anesthesia. The secondary outcomes were vital signs, incidence of adverse reactions, and Neonatal Apgar scores.

### Statistical analysis

SPSS 23.0 was used for data statistics in the current study. Side effects were expressed as number (%), and comparisons was expressed by χ2 test. Baseline characteristics, vital signs and operative characteristics were expressed as mean ± standard deviation (x ± SD) and analyzed using independent sample t test. The remaining measures were expressed as x ± SD and analyzed using Mann-Whitney U test. *P* < 0.05 was considered statistically significant.

## Results

**Fig. 1:**
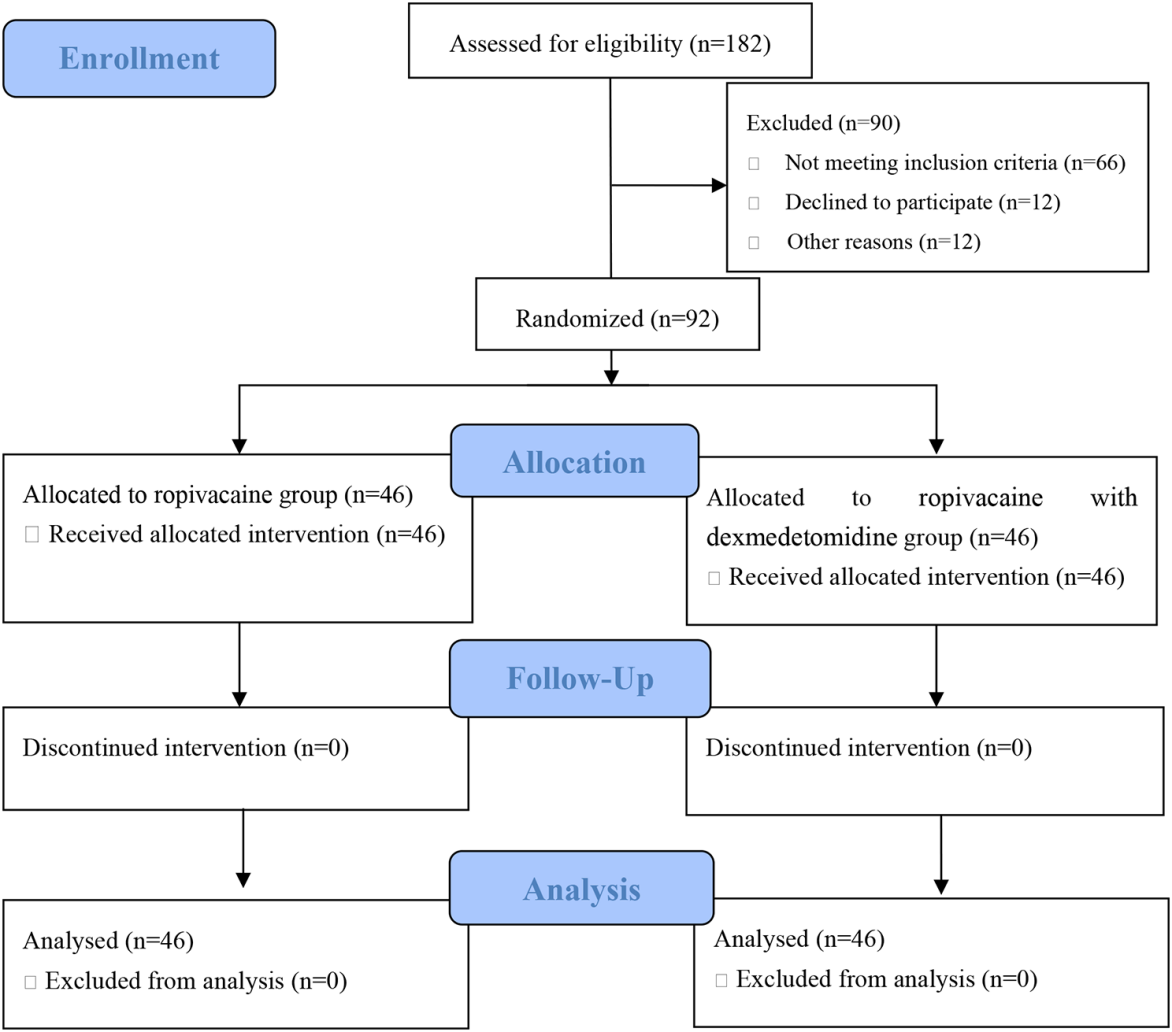
Flow diagram of study

Ninety-two parturients who were scheduled for elective caesarean section under EA were randomized to receive either epidural 90 mg ropivacaine with 0.5 µg/kg dexmedetomidine or 90 mg ropivacaine alone, and all parturients successfully completed the study (Fig. 1). Demographics of parturients and intraoperative characteristics are outlined in Table 1. The demographic data, initial vital signs, intraoperative measures were similar between two groups. The NRS were significantly lower in Group RD compared to the Group R at T2, T3, and T4 (Table 1).

**Table 1:** Baseline characteristics and intraoperative characteristics

	Group R (*n* = 46)	Group RD (*n* = 46)	F/Z	*p*-value
			F	
Average age (years)	30.5 ± 3.7	29.8 ± 3.3	0.338	0.563
Weight (kg)	67.6 ± 8.4	69.2 ± 9.4	0.188	0.665
Height (cm)	159.4 ± 5.8	158.4 ± 5.0	1.659	0.201
Gestation(weeks)	38.6 ± 1.0	39.0 ± 1.1	0.003	0.956
Initial vital signs				
Systolic BP (mmHg)	115.7 ± 10.3	117.6 ± 10.0	0.360	0.550
Diastolic BP (mmHg)	72.7 ± 9.4	72.6 ± 7.8	2.694	0.104
Heart rate (beats/min)	84.5 ± 13.4	85.8 ± 13.1	0.005	0.941
Intraoperative measures				
Length of surgery (min)	37.5 ± 6.3	39.3 ± 6.8	0.216	0.643
Blood loss (ml)	251.0 ± 61.5	281.5 ± 62.7	0.124	0.725
Urine volume (ml)	81.3 ± 51.5	104.1 ± 78.5	3.115	0.081
			Z	
Intraoperative pain (NRS)				
T2	1.96 ± 1.69	0.96 ± 1.40	-3.017	0.003
T3	1.20 ± 1.39	0.20 ± 0.62	-4.559	< 0.001
T4	1.43 ± 1.47	0.43 ± 1.00	-3.973	< 0.001
Sufentanil consumption (µg)	0.43 ± 1.42	0.11 ± 0.737	-1.372	0.170

Data are expressed as mean ± SD; statistical analysis was conducted using independent-samples t test or Mann-Whitney U test; Group R, ropivacaine group; Group RD, ropivacaine with dexmedetomidine group; BP, blood pressure; T2, fetal delivery; T3, uterus suture; T4, peritoneal closure; *p* < 0.05 was considered statistically significant

Sedation score between Group R and Group RD was similar at T0 (*P* > 0.05) while it was significantly higher in Group RD than that in Group R at T1, T2, T3 and T4 (*P* < 0.001). No parturient has experienced sedation score of 4 and above (Table 2).

**Table 2:** Comparison of intraoperative Ramsay Sedation Scale between the two groups

	Group R (*n* = 46)	Group RD (*n* = 46)	Z	*p*-value
T0	2.00 ± 0.00	2.02 ± 0.15	-1.000	0.317
T1	2.09 ± 0.29	2.78 ± 0.51	-6.309	< 0.001
T2	1.93 ± 0.25	2.26 ± 0.54	-3.590	< 0.001
T3	1.98 ± 0.15	2.57 ± 0.24	-5.839	< 0.001
T4	1.96 ± 0.21	2.65 ± 0.57	-6.309	< 0.001

Data are expressed as mean ± SD; statistical analysis was conducted using Mann-Whitney U test; Group R, ropivacaine group; Group RD, ropivacaine with dexmedetomidine group; T0, before anesthesia; T1, 15 min after EA; T2, fetal delivery; T3, uterus suture; T4, peritoneal closure. Ramsay Sedation Scale: 1, patient anxious and agitated or restless or both; 2, patient co-operative, orientated, and tranquil; 3, drowsiness but can respond to instructions; 4, brisk response; 5, a sluggish response; and 6, no response. *p* < 0.05 was considered statistically significant

There was no significant difference between Group R and Group RD regarding any intraoperative side effect and Apgar scores at 1–5 min after fetal delivery. The overall satisfaction with anesthesia was comparable between two groups (Table 3).

**Table 3:** Comparison of side effects, Apgar scores, and satisfaction with anesthesia between the two groups

	Group R (*n* = 46)	Group RD (*n* = 46)	χ^2^/Z	*p*-value
Hypotension, n (%)	15(32.6)	19(41.3)	0.756	0.388
Bradycardia, n (%)	7(15.2)	6(13.0)	0.090	0.765
nausea or vomiting, n (%)	14(30.4)	9(19.6)	1.449	0.229
1 min Apgar scores, mean ± SD	9.78 ± 0.417	9.70 ± 0.511	-0.769	0.442
5 min Apgar scores, mean ± SD	10.00 ± 0.000	10.00 ± 0.000	0.000	1.000
Satisfaction, mean ± SD	4.68 ± 0.702	4.82 ± 0.476	-0.628	0.530

Data are expressed as a number (%) or mean ± SD; statistical analysis was conducted using χ2 Test or Mann-Whitney U test; Satisfaction with anesthesia was assessed by using a five-point Likert scale: 1, very dissatisfied; 2, dissatisfied; 3, averagely satisfied; 4, satisfied; and 5, very satisfied; *p* < 0.05 was considered statistically significant

**Fig. 2:**
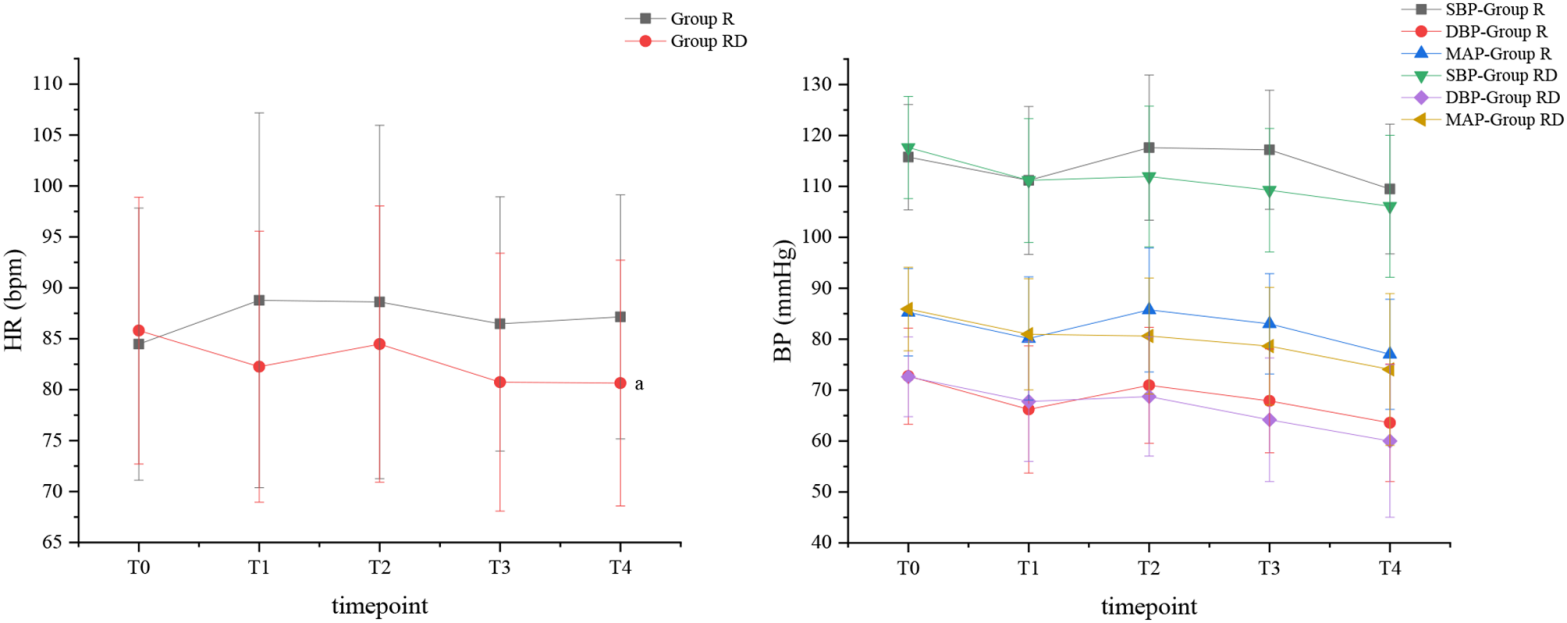
BP and HR between the two groups over study timepoints; Data are expressed as mean ± SD; BP, blood pressure; SBP, systolic blood pressure; DBP, diastolic blood pressure; MAP, mean arterial pressure; HR, heart rate; a *p* < 0.05 compared with Group R

There was no significant difference in SBP, DBP, and MAP between the two groups at the observed timepoint. HR of the two groups was comparable except at T4 (6.5 [95% CI, 0.7 to 12.3]; *P* < 0.001). (Fig. 2)

## Discussion

In this prospective, randomized, double-blind, placebo-controlled study, we observed that adding 0.5 µg/kg dexmedetomidine to ropivacaine for epidural anesthesia in parturients improved intraoperative analgesia significantly. Additionally, it enhanced the sedation level of parturients without inducing excessive sedation or other side effects. However, despite these benefits of epidural dexmedetomidine, overall satisfaction with anesthesia did not show a significant improvement.

Anesthesia plays an important role in surgery. Good anesthetic practice is composed of adequate analgesia, appropriate sedation, satisfying surgical requirements and rare side effect, thus contributes to good patient satisfaction. Nowadays, most cesarean sections are conducted under neuraxial anesthesia of which EA is one of the most popular anesthesia techniques, due to it’s simple, well controllable and cost effective [23]. However, many parturients (around 45-90%) didn’t get completely analgesia and high comfort during surgery as a possible consequence of incomplete blockade of large nerve roots such as L5, S1 and S2 [5], which may result in serious visceral pain when surgeon’s handling intraperitoneal organs or uterine contraction [6]. Besides, unlike other surgeries, there exists contradiction between sedation and good mother-baby contact in obstetric settings [15], thus parturient requires more suitable sedation level.

Previous studies have investigated the effect of applying a low dose of opioids (such as fentanyl) added to local anesthetics for neuraxial anesthesia, to enhance the neural block [24, 25]. Although anesthetic effect was improved, neuraxial opioids can also lead to serious side effects [7, 8], and parturients still didn’t benefit from appropriate sedation. Neuraxial dexmedetomidine mediates sedative, anxiolytic, analgesic and sympatholytic effect via central and peripheral α2-adrenergic receptor [18, 26], which may fits well the good anesthetic practice requirements as adjuvant drug to local anesthetic in cesarean section under EA.

The NRS is a common tool for the evaluation of the patient’s subjective feeling of the present pain intensity [21]. Dexmedetomidine epidural anesthesia could regulate the synthesis and release of catecholamines, reduce oxidative stress and attenuate inflammation which could alleviate pain [18, 27–30]. Yang et al. [31] investigated the effectiveness and safety of epidural injection of 0.75% ropivacaine (12 ml) and morphine (2 mg) combined with dexmedetomidine (0.5 µg/kg) for cesarean surgery. They found that NRS scores of visceral pain during operation were lower in group with dexmedetomidine than that without it. In our study, epidural ropivacaine with dexmedetomidine was more effective against intraoperative pain than ropivacaine alone (*P* < 0.05), which may be attributable to the aforementioned mechanism. Although the result was statistically significant, as small as a 1.0 difference in NRS pain score may not indicate a clinically meaningful difference [32]. In addition, supplemental analgesia of sufentanil was comparable between two groups at the present study, which was different from the report of Salem, and Moustafa et al. [33] who investigated the analgesic effect of epidural dexmedetomidine by adding 0.5 µg/kg dexmedetomidine to epidural bupivacaine and fentanyl in parturients undergoing elective cesarean section using combined spinal-epidural anesthesia for less additional intraoperative fentanyl consumption was found in the group of dexmedetomidine. This discrepancy could be due to fentanyl administered together with dexmedetomidine, for they can act synergistically to analgesic effect [33]. It is still not clear the analgesic effect, regarding pain NRS and requirement for supplemental analgesia, of a higher dose of epidural dexmedetomidine on parturients undergoing cesarean section.

When parturients undergo cesarean section under EA, they can stay awake, achieve early family contact and early food intake, which maintain the popularity of this anesthesia techniques [10]. However, the benefits mentioned above do come at a cost. The surgery procedure could easily result in parturients psychological stress which can further progress to anxiety and hyperalgesic responses [10, 34]. Patient satisfaction with sedation has been investigated widely and is generally very high [35, 36]. Therefore, it is very important to offer sedation in obstetric anesthesia. However, of equal importance is the maternal-neonatal contact immediately after birth, which can be affected by deep sedation. Thus, moderate or conscious sedation is more appropriate, let alone it’s much more cost efficient and safer than deep sedation. Dexmedetomidine acts through eliminating the inhibitory effect of the locus coeruleus on basal forebrain γ -amino butyric acid to produce sedative and hypnotic effects, from which patients are easily aroused [37]. At present study, the sedation level of parturients with dexmedetomidine was significantly higher than those without it. Gratifyingly, no parturient was experiencing sedation level of score ≥ 4 at the time of fetus delivery due to conscious sedation of dexmedetomidine which guaranteed the mother-baby bonding.

Satisfaction is a multidimensional concept, with influencing factors spanning physiological, psychological, and social aspects. Maternal satisfaction is associated with factors such as pain, sedation, and occurrence of adverse reactions [38, 39]. In our study, although dexmedetomidine significantly reduced maternal pain scores, as previously mentioned, a 1.0 difference in NRS pain score may not necessarily hold clinical significance, thus the alleviation of pain may have limited impact on satisfaction. Furthermore, dexmedetomidine improved maternal sedation levels, yet none of the parturients experienced excessive sedation in this study; therefore, the dexmedetomidine used did not adversely affect maternal-infant contact, making the sedative effect negligible in influencing satisfaction. Additionally, the occurrence rates of adverse reactions were similar between the two groups of parturients. These factors may explain the close similarity in satisfaction between the two groups of parturients.

It needs to be noted the potential hypotension and bradycardia associated with dexmedetomidine [33, 40]. In our study, although HR in Group RD was only significantly lower compared to Group R at T4, the incidence of hypotension and bradycardia was similar, which indicated the safety of the dexmedetomidine dosage used in our study, compared to a higher dose of dexmedetomidine 1.5 µg/kg that significantly decreased MAP and HR [41]. The relationship between pain and BP is poorly understood [42], While it is widely recognized that pain can raise BP through increasing sympathetic nerve activity. In our study, parturients in both groups experienced a slight decreasing trend of BP over the observational timepoints except for the timepoint of fetal delivery (T2) when the BP of parturients in Group R significantly increased compared to T1 (difference of MAP mean: 5.609 [95% CI, 0.755 to 10.462]; *P* = 0.013). The BP of Group RD was relative stable between T1 and T2 which also demonstrated the analgesic effect of dexmedetomidine from this perspective. We found no differences in Apgar scores between the two groups. This similarity also reflected the safety of epidural dexmedetomidine because of hemodynamic stability on the one hand, and because of its lipophilicity thus it easily retained in the placental tissue and transferred little to the fetus on the other hand [33].

The current study has several limitations. Firstly, some of the data collected was observational rather than based on laboratory indicators, which may have introduced documentation bias. Secondly, we were unable to measure the blood concentration of dexmedetomidine in both maternal and infant subjects due to technological limitations.

## Conclusion

Epidural dexmedetomidine of 0.5 µg/kg added slightly extra analgesic effect to ropivacaine in EA for cesarean section. The sedation of 0.5 µg/kg epidural dexmedetomidine did not cause mother-baby bonding deficit. Satisfaction with anesthesia wasn’t significantly improved by epidural dexmedetomidine of 0.5 µg/kg. No additional side effect allows larger dose of epidural dexmedetomidine attempt.

### Electronic supplementary material

Below is the link to the electronic supplementary material.

Supplementary Material 1

Supplementary Material 2

## Data Availability

The datasets used and analyzed during the current study are available from the corresponding author upon reasonable request.

## Abbreviations

EA: epidural anesthesia
GA: general anesthesia
ASA: American Society of Anesthesiologists
NIBP: non-invasive blood pressure
NRS: numerical rating scale
MAP: mean arterial pressure
HR: heart rate
