# Supplementary material for: Effect of epidural dexmedetomidine in single-dose combined with ropivacaine for cesarean section
Source: BMC Anesthesiol. 2024 Apr 8;24:134. doi: 10.1186/s12871-024-02519-4 (PMC11000346; doi:10.1186/s12871-024-02519-4)
Supplement: Supplementary file 2 — Supplementary Material 2 [file 12871_2024_2519_MOESM2_ESM.docx]

**Project summary**

Our study, " **Epidural Infusion of Dexmedetomidine Improves the Experience of Primiparas Undergoing** **Cesarean Section with Epidural Anesthesia: A Randomized Double‑Blinded Controlled Study** " aims to explore the effects of 0.5μg/kg epidural dexmedetomidine combined with epidural anesthesia on parturients undergoing cesarean section. This research seeks to contribute valuable insights to the field of obstetric anesthesia by evaluating the analgesic and sedative properties of dexmedetomidine.

**Rationale:**

Current literature emphasizes the arousal sedation and analgesic effects of dexmedetomidine. This project addresses a critical research gap by specifically investigating the application of 0.5μg/kg epidural dexmedetomidine in combination with EA for cesarean section. The goal is to enhance analgesic efficacy without compromising maternal satisfaction or causing adverse effects.

**Objectives:**

Evaluate the additional analgesic effect of 0.5μg/kg epidural dexmedetomidine when combined with epidural ropivacaine during cesarean section. Examine the sedative impact of 0.5μg/kg epidural dexmedetomidine using the Ramsay Sedation Scale. Assess maternal satisfaction, incidence of adverse effects, and the potential for larger doses of epidural dexmedetomidine.

**Methods:**

Conducted as a randomized control trial, the study involves 92 parturients, randomly assigned to Group R (receiving epidural ropivacaine alone) or Group RD (receiving epidural ropivacaine with 0.5μg/kg dexmedetomidine). Primary outcomes include intraoperative NRS pain scores and Ramsay Sedation Scale assessments. Rigorous data collection includes comprehensive pre- and post-assessments, surveys, and meticulous observation.

**Populations:**

This study exclusively includes primiparas undergoing cesarean section, eliminating variability from previous epidural anesthesia experiences for a more precise evaluation of current outcomes.

**Time Frame:**

The research spans two academic semesters, allowing for an extensive analysis of immediate and sustained effects. A systematic timeline governs data collection, analysis, and result dissemination.

**Expected Outcomes:**

Anticipated outcomes involve the identification of the incremental analgesic benefits of 0.5µg/kg epidural dexmedetomidine in combination with ropivacaine for EA during cesarean section. Our findings demonstrate that this sedative dosage does not adversely affect mother-baby bonding or overall satisfaction. While satisfaction with anesthesia remains unaltered, the absence of significant side effects supports potential exploration of larger doses of epidural dexmedetomidine in future studies.

**General information**

- Protocol title: **Epidural Infusion of Dexmedetomidine Improves the Experience of Primiparas Undergoing Cesarean Section with Epidural Anesthesia: A Randomized Double‑Blinded Controlled Study**
- Protocol identifying number (if any), and date:**2020-555, 2020/10/14**
- Name and address of the sponsor/funder: **Funding Chongqing Science and Technology Bureau (cstc2018jscx-msybx002) and Chongqing Health Bureau (2018GDRC009, 2018 jstg011, 2019jstg003, 2021jstg045).**
- **Minghao Liu, (resident)** is responsible for conducting the research, and the address and telephone number of the research site are: **Department of Anesthesiology, the First Affiliated Hospital of Chongqing Medical University, Chongqing, PR China, 023-68811360**

**Rationale & background information**

Cesarean sections are commonly performed under general anesthesia (GA) or neuraxial anesthesia, with epidural anesthesia (EA) being a preferred technique. EA mitigates risks associated with GA, maintains positive early mother-child bonding, and has a more favorable impact on hemodynamics than spinal anesthesia(1–4). Despite these advantages, EA has limitations in providing sufficient visceral pain relief during surgery(5) and exhibits a slow onset of action. Combining adjuvants, such as sufentanil, with local anesthetics has become a common approach to enhance EA(6). However, neuraxial opioids present side effects, including pruritus, nausea, vomiting, and potentially life-threatening respiratory depression(7,8).

Sedation offers a promising avenue to reduce opioid reliance during regional anesthesia, lowering the incidence of postoperative nausea and vomiting(9). Additionally, sedation contributes to higher patient satisfaction and increased acceptance of regional anesthesia(10,11). In obstetric settings, early skin-to-skin contact between mother and newborn has numerous benefits, including a reduction in postpartum bleeding rates(12), the promotion of oxytocin and endorphin release(13,14), enhancing maternal mood(14). However, inappropriate sedation may impact this critical skin-to-skin contact(15).

Dexmedetomidine, a highly selective α2 agonist, exhibits arousal sedation, analgesic, and anti-sympathetic effects without causing respiratory depression or other opioid-associated drawbacks(1,16). Previous studies have demonstrated the efficacy of intrathecally administered dexmedetomidine in combination with local anesthetics in improving intraoperative anesthesia for neuraxial anesthesia and enhancing maternal satisfaction after cesarean section(17–19). However, the specific impact of combining epidural dexmedetomidine with local anesthetic in cesarean sections under epidural anesthesia remains insufficiently understood.

This prospective, randomized, double-blind controlled study aims to investigate the effectiveness of epidural dexmedetomidine in combination with ropivacaine on parturients undergoing cesarean section with epidural anesthesia. The study addresses gaps in knowledge concerning the optimal use of dexmedetomidine in this specific context, contributing valuable insights to improve anesthesia outcomes for cesarean deliveries.

**References (of literature cited in preceding sections)**

1. Siddik-Sayyid S, Zbeidy R. Practice guidelines for obstetric anesthesia. Vol. 19, Middle East Journal of Anesthesiology. 2008. 1291–1303 p.

2. Carrie LES. Extradural, spinal or combined block for obstetric surgical anaesthesia. Br J Anaesth. 1991;66(4):528.

3. Zhou M, Liu W, Peng J, Wang Y. Impact of propofol epidural anesthesia on immune function and inflammatory factors in patients undergoing gastric cancer surgery. Am J Transl Res. 2021;13(4):3064–73.

4. Xu W, Drzymalski DM, Ai L, Yao H, Liu L, Xiao F. The ED50 and ED95 of Prophylactic Norepinephrine for Preventing Post-Spinal Hypotension During Cesarean Delivery Under Combined Spinal-Epidural Anesthesia: A Prospective Dose-Finding Study. Front Pharmacol. 2021;12(July):1–7.

5. Alahuhta S, Kangas‐Saarela T, Hollmén AI, Edström HH. Visceral pain during caesarean section under spinal and epidural anaesthesia with bupivacaine. Acta Anaesthesiol Scand. 1990;34(2):95–8.

6. Lu Q, Dong CS, Yu JM, Sun H, Sun P, Ma X, et al. The dose response of sufentanil as an adjuvant to ropivacaine in cesarean section for relief from somato-visceral pain under epidural anesthesia in parturients with scarred uterus. Med (United States). 2018/09/22. 2018;97(38):e12404.

7. Armstrong S, Fernando R. Side Effects and Efficacy of Neuraxial Opioids in Pregnant Patients at Delivery: A Comprehensive Review. Drug Saf. 2016/02/03. 2016;39(5):381–99.

8. Melo H, Basso L, Iftinca M, MacNaughton WK, Hollenberg MD, McKay DM, et al. Itch induced by peripheral mu opioid receptors is dependent on TRPV1-expressing neurons and alleviated by channel activation. Sci Rep [Internet]. 2018;8(1):1–9. Available from: http://dx.doi.org/10.1038/s41598-018-33620-7

9. Avramov MN, White PF. Use of alfentanil and propofol for outpatient monitored anesthesia care: Determining the optimal dosing regimen. Anesth Analg. 1997;85(3):566–72.

10. Höhener D, Blumenthal S, Borgeat A. Sedation and regional anaesthesia in the adult patient. 2007/12/12. Vol. 100, British Journal of Anaesthesia. 2008. p. 8–16.

11. Li T, Ye Q, Wu D, Li J, Yu J. Dose-response studies of Ropivacaine in blood flow of upper extremity after supraclavicular block: A double-blind randomized controlled study. BMC Anesthesiol. 2017;17(1):1–7.

12. Saxton A, Fahy K, Rolfe M, Skinner V, Hastie C. Does skin-to-skin contact and breast feeding at birth affect the rate of primary postpartum haemorrhage: Results of a cohort study. Midwifery [Internet]. 2015;31(11):1110–7. Available from: https://www.sciencedirect.com/science/article/pii/S026661381500203X

13. Govoni L, Ricchi A, Molinazzi MT, Galli MC, Putignano A, Artioli G, et al. Breastfeeding pathologies: Analysis of prevalence, risk and protective factors. Acta Biomed. 2019;90:56–62.

14. Er M, Bergman N, Gc A, Medley N. Moore ER et al. Early skin-to-skin contact formothers and their healthy newborn infants. Cochrane Database of Systematic Reviews 2016, Issue 11. Art. No.: CD003519. DOI: 10.1002/14651858.CD003519.pub4. Cochrane Database Syst Rev [Internet]. 2016;(11). Available from: https://www.cochranelibrary.com/cdsr/doi/10.1002/14651858.CD003519.pub4/epdf/standard

15. Liu S, Peng P, Hu Y, Liu C, Cao X, Yang C, et al. The effectiveness and safety of intravenous dexmedetomidine of different concentrations combined with butorphanol for post-caesarean section analgesia: A randomized controlled trial. Drug Des Devel Ther. 2021;15:689–98.

16. Conti G, Ranieri VM, Costa R, Garratt C, Wighton A, Spinazzola G, et al. Effects of dexmedetomidine and propofol on patient-ventilator interaction in difficult-to-wean, mechanically ventilated patients: A prospective, open-label, randomised, multicentre study. Crit Care [Internet]. 2016;20(1):1–8. Available from: http://dx.doi.org/10.1186/s13054-016-1386-2

17. Bi YH, Wu JM, Zhang YZ, Zhang RQ. Effect of Different Doses of Intrathecal Dexmedetomidine as an Adjuvant Combined With Hyperbaric Ropivacaine in Patients Undergoing Cesarean Section. Front Pharmacol. 2020;11:342.

18. Bi YH, Cui XG, Zhang RQ, Song CY, Zhang YZ. Low dose of dexmedetomidine as an adjuvant to bupivacaine in cesarean surgery provides better intraoperative somato-visceral sensory block characteristcs and postoperative analgesia. Oncotarget. 2017;8(38):63587–95.

19. Shin DW, Kim Y, Hong B, Yoon SH, Lim CS, Youn S. Effect of fentanyl on nausea and vomiting in cesarean section under spinal anesthesia: a randomized controlled study. J Int Med Res. 2019;47(10):4798–807.

**Study goals and objectives**

To enhance the efficacy and safety of epidural anesthesia in cesarean sections by investigating the combined use of epidural dexmedetomidine with ropivacaine, thereby addressing the limitations of traditional neuraxial techniques.

**Study design**

This is a prospective, randomized, double-blind controlled study. Parturients who are scheduled for elective caesarean section under EA between December 2020 and July 2021 were eligible for this study. The inclusion criteria for the study were as follows: age between 18 and 39 years old; singleton pregnancy; American Society of Anesthesiologists (ASA) physical status I or II; ≥ 37 weeks’s gestation. The exclusion criteria of the study were as follows: EA is contraindicated; organ dysfunction such as hypertension, cardiopulmonary disease, placenta previa, fetal distress in utero, and cardiac conduction or rhythm abnormalities; allergy or intolerance to one of the study medications, chronic analgetic use for longer than 3 months; any previous EA or abdominal surgery.

**Methodology**

**Randomization and Masking:**

- Parturients randomly assigned (1:1 ratio) to Group R (epidural 90mg ropivacaine) or Group RD (90mg ropivacaine with 0.5μg/kg dexmedetomidine).
- Randomization accessible only to the researcher preparing medications.
- Blinding maintained for researchers, surgeons, anesthesiologists, nurses, midwives, and participants until study completion.

**Anesthesia Procedure:**

- Epidural puncture at 2-3 lumbar interspace in right lateral decubitus position.
- Epidural catheter inserted 4cm cephalad; test dose of 3 mL 1% lidocaine administered.
- Group-specific medication administered based on randomization.
- Effectiveness of EA defined as bilateral T6-S5 or above sensory block within 30min.

**Monitoring and Interventions:**

- Standard monitoring upon arrival in the operating room.
- Intraoperative assessments, including pain severity (NRS), sedation (Ramsay Sedation Scale), vital signs, and side effects recorded.
- Interventions for pain and sedation as necessary.
- Timepoints defined (T0 to T4) for specific assessments during the procedure.

**Safety considerations**

**Definition and management of hypotension:**

Definition: Mean Arterial Pressure (MAP) < 60mmHg or > 20% decline from baseline BP.

Management: Intravenous administration of 50μg phenylephrine, repeated if necessary.

**Definition and management of bradycardia:**

Definition: Heart Rate (HR) < 60 beats/min.

Management: If no hypotension is present, intravenous administration of 0.25mg atropine; if bradycardia is present with hypotension, then 6mg ephedrine is given.

**Definition and management of intraoperative respiratory depression:**

Definition: Respiratory rate less than or equal to 8 breaths/min or SpO2 < 95%.

Management: Assisted ventilation is used for treatment.

**These measures aim to ensure the timely management of potential safety issues during the surgical procedure, safeguarding the safety of research participants.**

**Follow-up**

The data we collected were primarily obtained intraoperatively, and at three months postoperatively, we conducted telephone follow-ups. The follow-up included assessments of infant health, maternal health, the presence of anesthesia-related complications, and satisfaction with the anesthesia.

**Data management and statistical analysis**

**Data Collection:**

- Demographics and intraoperative measures collected.
- Data points include NRS pain scores, Ramsay Sedation Scale, NIBP, HR, side effects, and neonatal Apgar scores.
- Satisfaction with anesthesia assessed at 3 months post-hospital discharge via telephone follow-up.

**Statistical Analysis:**

- SPSS 23.0 used for data analysis.
- Presentation of side effects as percentages; comparisons conducted using χ2 test.
- Baseline and operative characteristics presented as mean ± SD; analyzed with t-test or Mann-Whitney U test.
- Significance considered at P < 0.05.

**Quality assurance**

The Ethics Committee supervised the entire trial process and appointed a senior anesthesiologist to oversee the anesthesia procedure in case of emergencies.

**Expected outcomes of the study**

Anticipated outcomes involve the identification of the incremental analgesic benefits of 0.5µg/kg epidural dexmedetomidine in combination with ropivacaine for EA during cesarean section. Our findings demonstrate that this sedative dosage does not adversely affect mother-baby bonding or overall satisfaction. While satisfaction with anesthesia remains unaltered, the absence of significant side effects supports potential exploration of larger doses of epidural dexmedetomidine in future studies.

**Duration of the project**

The study was conducted from between December 2020 and July 2021, with a follow-up period of three months for each participant after the surgery.

**Problems anticipated**

Not applicable

**Project management**

**Minghao Liu** collected, analyzed and interpreted all data and was a major contributor in writing the manuscript. **Xuezi Chen** enrolled the patients and guided informed

consent. **Dan Guo** conducted statistics and analysis of data, guided paper writing

and provided financial and technical support.

**Ethics**

This study was accredited by the Ethics Committee of The First Affiliated Hospital of Chongqing Medical University. Participants were well-informed of the study protocol and written informed consent was taken from all the participants present in the study.

**Informed consent forms**

**background**：

Cesarean sections often use epidural anesthesia (EA) due to its advantages, but it has limitations. Combining adjuvants like sufentanil helps, but opioids have side effects. Sedation is promising for reducing opioid use and improving patient satisfaction. Early skin-to-skin contact is vital in obstetrics, but inappropriate sedation can impact it. Dexmedetomidine, an α2 agonist, has proven effective in intrathecal administration with local anesthetics for cesarean sections. However, combining epidural dexmedetomidine with local anesthetic in this context needs more understanding.

Our study aims to investigate the effectiveness of epidural dexmedetomidine with ropivacaine in cesarean sections under epidural anesthesia. Addressing knowledge gaps, it provides insights into optimizing dexmedetomidine use, contributing valuable information for better anesthesia outcomes in cesarean deliveries.

**research process：**

Parturients were randomly assigned in a 1:1 ratio to either Group R (epidural 90mg ropivacaine) or Group RD (90mg ropivacaine with 0.5μg/kg dexmedetomidine). The anesthesia procedure included epidural puncture at the 2-3 lumbar interspace in the right lateral decubitus position, with an epidural catheter inserted 4cm cephalad, followed by the administration of a test dose of 3 mL 1% lidocaine. Group-specific medication was then administered based on the randomization. Monitoring and interventions involved standard procedures upon arrival in the operating room, intraoperative assessments, including pain severity (NRS), sedation (Ramsay Sedation Scale), vital signs, and the recording of side effects. Interventions for pain and sedation were implemented as necessary, with specific timepoints (T0 to T4) defined for assessments during the procedure.

**Potential Benefits of the Study：**

You will receive one-on-one anesthesia services, and we will cover the cost of your anesthesia medications. According to previous literature, this is expected to result in an improved anesthesia outcome for you.

**Risks and Discomforts during the Study：**

The dose of dexmedetomidine used in this study falls within a safe and effective range. Adverse risks during the procedure may include bradycardia, hypertension or hypotension, and intolerable pain; postoperatively, there may be risks such as bleeding at the anastomotic site and surgical site infection. The occurrence of adverse reactions is not significantly related to participation in this study; even without participating, these adverse reactions may still occur. Adverse reactions are assessed based on the following criteria, and appropriate treatment is provided even if they are unrelated to the trial:

Definitely related: There is evidence of trial intervention; the timing of the adverse event is credible and correlates with the trial intervention; the trial intervention is a more plausible explanation for the occurrence of the adverse event than other reasons; a positive response to stopping the intervention; the pattern of adverse events is consistent with prior knowledge of this type of intervention.

Possibly related: There is evidence of trial intervention; the timing of the adverse event is credible and correlates with the trial intervention; the adverse event may be caused by the trial intervention or other reasons; a positive response to stopping the intervention.

Possibly unrelated: There is evidence of trial intervention; the adverse event is more likely caused by other reasons; a negative or ambiguous response to stopping the intervention.

Definitely unrelated: The patient did not receive trial intervention; or the timing of the adverse event is not credible in relation to the trial intervention; or there are other significant reasons for the adverse event.

Uncertain: If a suspected adverse reaction cannot be evaluated using the above four criteria, it is deemed uncertain. Close observation and monitoring of patients are essential during the perioperative period. Throughout the study, Associate Professor Wang Bin from the Department of Anesthesiology at the First Affiliated Hospital of Chongqing Medical University is responsible for medical supervision and the safety of subjects. In case of adverse events, detailed records of the specific circumstances and relevant measures taken should be made, and patient data should be preserved.

**Privacy Issues:**

If you decide to participate in this study, all relevant data, including identity information and complete personal medical records, will be treated as confidential documents and processed accordingly. The data verification process will be conducted with the utmost confidentiality. Members of the research team will keep confidential any related information and results from the study, and unless you agree, any information that can identify you will not be disclosed to anyone outside the research team. After the trial is completed, all case report forms will be filled out, reviewed, and signed by the researchers and monitors according to the data management requirements of this protocol. Along with the "Informed Consent Form," they will be handed over to a designated person in the department for storage. All research-related documents must be retained for at least 15 years after the study concludes. After this time period, with the consent of the First Affiliated Hospital of Chongqing Medical University, we will proceed with the destruction of these materials. Additionally, when the results of this study are published, we will also strictly maintain the confidentiality of your relevant information.

**Costs and Compensation:**

This study involves data collection under the current commonly used and more effective treatment methods, without interfering with your other treatments, and will not incur additional medical expenses. The costs of various tests and assessments required for this study will be covered by our research team. If you experience any harm due to participating in this study, such as severe adverse reactions, you will receive free treatment for the severe adverse reactions and corresponding compensation. The treatment and compensation costs will be provided by our research team.

**Voluntary Withdrawal:**

As a participant, you have the right to be informed about information and progress related to this study, and you can voluntarily decide whether to (continue) participate or not. After joining, regardless of whether harm occurs or how serious it is, you can choose to notify the researcher to withdraw from the study at any time without any reason. Your data will not be included in the study results, and your medical treatment and rights will not be affected. If continuing to participate in the study poses a serious risk of harm to you, the researcher will terminate the study. However, during the study, please provide truthful information about your medical history and current physical condition; inform the research doctor of any discomfort you experience during this study; do not take restricted drugs, foods, etc.; inform the research doctor if you have recently participated in or are currently participating in other studies. If you do not adhere to the research plan or if any harm or other reasons related to the study occur, the research doctor may terminate your continued participation in this study.

**Contact Information:**

If you have any questions related to this study, or if you experience any discomfort or injury during the research process, or if you have questions about the rights of participants in this study, you can contact us. Contact person: Minghao Liu, Phone: 17629720320.

**Benefits Sharing After the Trial:**

When the study is completed and proves to be safe and effective, there are no economic benefits.

**Informed Consent Signature:**

I have read this informed consent form, and my doctor, [Doctor's Name], has provided detailed explanations about the purpose, content, risks, and benefits of this clinical trial. They have answered all the questions I asked. I understand this clinical study, and I voluntarily agree to participate in it.

Subject's Signature: ____________________ Date: Year _____ Month _____ Day _____ Researcher's Signature: _________________ Date: Year _____ Month _____ Day _____ (Note: If the subject is illiterate, the witness's signature is required. If the subject lacks the capacity to act, the consent of the legal representative is required.)

**知情同意书**

**研究背景介绍：**

尊敬的患者（受试者）：

您好！

由于您选择择期硬膜外剖宫产。您将被邀请参加一项由重庆医科大学附属第一医院麻醉科主持的研究。这是为了探究在硬膜外麻醉剖宫产的局麻药中辅用不同剂量的右美托咪定对抑制牵拉反应有效且较为安全的适宜剂量，为临床提供参考意见而进行的一项研究，它将历时6个月时间。

本知情同意书提供给您一些信息以帮助您决定是否参加此项临床研究。您参加本项研究是自愿的。本次研究已通过本研究机构伦理审查委员会审查。如果你同意加入此项研究，请看下列说明：

请您仔细阅读，如有任何疑问请向负责该项研究的研究者提出。

**研究目的：**

剖宫产常采用硬膜外麻醉（EA）以其优点，但也存在局限。辅助剂如舒芬太尼有助于缓解，但阿片类药物有副作用。镇静有望减少阿片类药物的使用，提高患者满意度。产科中早期母婴皮肤接触至关重要，但不当的镇静可能影响它。右美托咪定，一种α2激动剂，通过与局麻药物联合腰麻已在剖宫产中证明其有效性。然而，在硬膜外麻醉的情境下联合右美托咪定和局麻药物尚需更深入了解。

我们的研究旨在探讨硬膜外右美托咪定联合罗哌卡因在硬膜外麻醉剖宫产中的有效性。弥补知识空白，为优化右美托咪定的使用提供见解，为改善剖宫产麻醉结果提供宝贵信息。

**研究过程和方法：**

产妇以1:1的比例随机分配到R组（硬膜外90毫克罗哌卡因）或RD组（90毫克罗哌卡因与0.5μg/kg右美托咪定）。麻醉程序包括在右侧卧位2-3腰椎间隙进行硬膜外穿刺，插入硬膜外导管至头侧4厘米，随后注射3毫升1%利多卡因的试验剂量。然后根据随机分组方案给予特定组别的药物。监测和干预包括患者抵达手术室后的标准程序，术中评估，包括疼痛强度（NRS），镇静程度（Ramsay镇静评分），生命体征，并记录副作用。根据需要实施疼痛和镇静的干预，为在手术过程中进行评估定义了特定的时间点（T0到T4）。

**研究可能的受益**：

您将受到一对一的麻醉医生的服务，我们将免除您的麻醉药品费用，根据既往文献报道，您的麻醉效果将更加好。

**研究风险与不适**：

本次研究所使用右美托咪定剂量均安全有效的剂量范围内。其次，研究中不良风险包括术中可能出现心动过缓、高血压或低血压、疼痛不能耐受，术后可能出现吻合口出血、手术部位感染等风险。不良反应的发生与是否参加本研究无明显关系，即使不参加本研究，这些不良反应亦可能发生。根据以下标准评定不良反应是否和试验有关，即使与试验无关，也需妥善处治。1）肯定有关：有给予试验干预证据，不良事件的出现与给予试验干预的时间顺序是可信的；不良事件的产生由试验干预解释较其他原因更合适；停止干预反应阳性；不良事件模式与既往对这种或这类干预的了解一致。2）可能有关：有给予试验干预证据；不良事件的出现与给予试验干预的时间顺序是可信的；不良事件的产生由试验干预导致，也可能由其他原因所致；停止干预反应阳性。3）可能无关：有给予试验干预证据；不良事件更可能由其他原因所致；停止干预反应阴性或模棱两可。4）肯定无关：患者未给予试验干预；或不良事件的出现与给予试验干预的时间顺序不可信；或有其他显著的原因可导致不良事件。5）不确定：可疑不良反应不能用以上四个标准评价，则认定为不能确定。围术期必须对患者进行严密的观察与监测。研究过程中，由重庆医科大学附属第一医院麻醉科王彬副教授负责医疗监督及整个研究过程中受试者的安全，如发生不良事件，应详细记录不良事件发生的具体情况及相关处理措施，并保存患者资料。

**隐私问题：**

如果您决定参加本项研究，如果您决定参加本项研究，您的全部相关数据，包括身份识别信息和全部个人医学资料应视作保密文件，按保密文件处理。数据核对程序应按最严格保密方式进行。研究工作组成员应对研究中的任何相关信息和结果保密，除非您本人认可，否则任何可以识别您身份的信息将不会透露给研究小组以外的成员。试验完成后，全部病例报告表经研究人、监察员按本方案数据管理的要求填写、审核和签字，连同“知情同意书”交科室领导指定专人保存。全部研究相关文件必须在研究结束后保存至少15年。在这一时限到期后，我们在征得重庆医科大学第一附属医院同意后销毁。此外这项研究结果发表时，我们也将对您的相关信息进行严格保密。

**费用和补偿：**

此研究是在予以目前常用的疗效较好的治疗方法的情况下进行数据收集，不干涉您的其他治疗，不会额外增加医疗费用。本研究所需的各项测试、评估费用由本课题组提供。如果您因参与这项研究而受到伤害：如因参与这项研究而出现的相关严重不良反应，您可以免费接受严重不良反应的治疗和相应的补偿。治疗和补偿费用由本课题组提供。

**自由退出：**

作为受试者，您可随时了解与本研究有关的信息资料和研究进展，自愿决定（继续）参加还是不（继续）参加。参加后，无论是否发生伤害，是否严重，您可以选择在任何时候不需要任何理由通知研究者要求退出研究，您的数据将不纳入研究结果，您的任何医疗待遇与权益不会因此而受到影响。如果继续参加研究，会对您造成严重的伤害，研究者也将会终止研究的进行。

但在参加研究期间，请您提供有关自身病史和当前身体状况的真实情况；告诉研究医生自己在本次研究期间所出现的任何不适；不得服用受限制的药物、食物等；告诉研究医生自己在最近是否曾参与其他研究，或目前正参与其他研究。如果因为您没有遵守研究计划，或者发生了与研究相关的损伤或者有任何其它原因，研究医师可以终止您继续参与本项研究。

**联系方式：**

如果您有与本研究有关的问题，或您在研究过程中发生了任何不适与损伤，或有关于本项研究参加者权益方面的问题，您可以与我们联系，联系人：刘銘浩 电话：17629720320**。**

**试验后利益分享：**

当研究结束且研究证明安全有效时，不存在经济利益。

**知情同意签字：**

我已经阅读了本知情同意书，并且我的医生 已经将此次临床试验的目的、内容、风险和受益情况向我作了详细的解释说明，对我询问的所有问题也给予了解答，我对此项临床研究已经了解，我自愿参加本项研究。

受试者签名： 日期： 年 月 日

研究者签名： 日期： 年 月 日

（注：如果受试者不识字时尚需见证人签名，如果受试者无行为能力时则需代理人同意）

**Research protocol: part 2**

**Budget**

**Personnel Costs (30% of Budget) - $1500：**

Principal Investigator (PI) Salary: $1000

Research Assistants (X): $500

**Medical Supplies and Drug Procurement (25% of Budget) - $1250**

Purchase of Investigational Drugs: $750

Lab Supplies: $500

**Data Management and Analysis (15% of Budget) - $750**

Database Software License: $500

Statistical Analysis Software: $250

**Ethics Committee Fees and Regulatory Compliance (5% of Budget) - $250**

Institutional Review Board (IRB) Submission Fees: $250

**Publication and Dissemination (5% of Budget) - $250**

Manuscript Preparation and Submission: $150

Conference Participation: $100

**Total Project Budget: $6000**

**Other support for the project**

Not applicable

**Collaboration with other scientists or research institutions**

Not applicable

**Links to other projects**

Not applicable

**Curriculum Vitae of investigators**

**Minghao Liu^1^, Xuezi Chen^2^, and Dan Guo^3*^**

^1^ Department of Anesthesiology, Chengdu Fifth People's Hospital (The Second Clinical Medical College, Geriatric Diseases Institute of Chengdu/Cancer Prevention and Treatment Institute of Chengdu, Affiliated Fifth People's Hospital of Chengdu University of Traditional Chinese Medicine), Chengdu, China.

^2^ Department of Anesthesiology, the First Affiliated Hospital of Chongqing Medical University, Chongqing, China

^3^ Department of Ultrasound Imaging, Chengdu Fifth People's Hospital (The Second Clinical Medical College, Geriatric Diseases Institute of Chengdu/Cancer Prevention and Treatment Institute of Chengdu, Affiliated Fifth People's Hospital of Chengdu University of Traditional Chinese Medicine), Chengdu, 611137, China; E-mail: 924251258@qq.com.

**Financing and insurance**

Not applicable
